# Oxygen Consumption and Acoustic Activity of Adult *Callosobruchus maculatus* (F.) (Coleoptera: *Chrysomelidae*: Bruchinae) during Hermetic Storage

**DOI:** 10.3390/insects9020045

**Published:** 2018-04-20

**Authors:** Anastasia W. Njoroge, Richard W. Mankin, Bradley W. Smith, Dieudonne Baributsa

**Affiliations:** 1 Department of Entomology, Purdue University, 901 W. State Street, West Lafayette, IN 47907, USA; annwanjiru608@gmail.com (A.W.N.); smit3000@purdue.edu (B.W.S.); 2 United States Department of Agriculture, Agricultural Research Service Center for Medical, Agricultural and Veterinary Entomology, 1700 SW 23rd Dr, Gainesville, FL 32608, USA; Richard.Mankin@ars.usda.gov

**Keywords:** cowpea weevil, detection, mortality, hermetic, postharvest

## Abstract

Acoustic monitoring was applied to consider hermetic exposure durations and oxygen levels required to stop adult *Callosobruchus maculatus* activity and economic damage on cowpea. A 15-d study was conducted with six treatments of 25, 50, and 100 *C. maculatus* adults in 500 and 1000 mL jars using acoustic probes inserted through stoppers sealing the jars. Acoustic activity as a result of locomotion, mating, and egg-laying was measured by identifying sound impulses with frequency spectra representative of known insect sounds, and counting trains (bursts) of impulses separated by intervals of <200 ms, that typically are produced only by insects. By the end of the first week of storage in all treatments, oxygen levels declined to levels below 4%, which has been demonstrated to cause mortality in previous studies. Concomitantly, insect sound burst rates dropped below an acoustic detection threshold of 0.02 bursts s^−1^, indicating that the insects had ceased feeding. Statistically significant relationships were obtained between two different measures of the acoustic activity and the residual oxygen level. Based on the experimental results, a simple equation can be used to estimate the time needed for oxygen to decline to levels that limit insect feeding damage and thus grain losses in hermetic storage containers of different insect population levels and various volumes.

## 1. Introduction

Cowpea (*Vigna unguiculata* (L.) Walp.) is an economically important indigenous protein source in the semi-arid tropical and subtropical world, namely Asia, Africa, southern Europe, and Central and South America [1]. It is particularly important in Sub-Saharan Africa compared to other regions, with consumption growing at the rate of 3.2% per annum between 1980 and 2009 [1]. Cowpea is severely damaged during storage by insect-pests such as *Callosobruchus maculatus* (Coleoptera: *Chrysomelidae*: Bruchinae), *Callosobruchus chinensis* (Coleoptera: *Chrysomelidae*: Bruchinae), and *Bruchidious atrolineatus* (Coleoptera: *Chrysomelidae*: Bruchinae) [2,3].

*Callosobruchus maculatus* is an important pest of stored legume grains including cowpea and widely distributed all over the continent [4]. It spreads with grain trade and greatly lowers the value of dried pulses in markets, especially in Sub Saharan Africa. Infestation usually starts in the field before harvest and proliferates to devastating levels during storage, depending on the methods of storage used. 

Pre-harvest practices that reduce field infestation of cowpea include crop management such as site selection, proper planting, and harvesting methods that make the habitat unfavorable for *C. maculatus* [5]. Post-harvest control measures for *C. maculatus* include but are not limited to fumigants and insecticides. Alternative non-chemical control methods include cold storage exposures, solarisation, storage with ash and other biorational products, bruchid resistant cultivars, controlled atmospheres, and hermetic storage [6,7,8,9,10,11]. Hermetically sealed containers such as metal silos and single or multiple plastic bagging control *C. maculatus* infestations through the accumulation of CO_2_ to lethal levels of >40% *v*/*v* and depletion of O_2_ to less than 2% *v*/*v* produced through insect metabolism [11,12,13,14]. In the recent past, there has been a rise in the number of hermetic storage technologies being sold in Sub Saharan Africa under various trade names, including Purdue Improved Crop Storage (PICS), AgroZ, Elite, Grain Pro, Metal Silos, and Zero-Fly [15].

The lethal action of low oxygen and oxygen-free atmospheres on postharvest insect pests during storage has been studied over many years [16,17,18,19,20]. Effectiveness of hermetic bags against *C. maculatus* has also been tested at the farm level [10,13]. The behavior of adults and other developmental stages of *C. maculatus* under low oxygen (hypoxia), and other conditions such as high carbon dioxide (hypercarbia) and low oxygen-high carbon dioxide (hypoxia/hypercarbia), has been investigated [19,21,22]. However, the behavioral activity associated with slowly declining oxygen levels during hermetic storage has been characterized only minimally. A recent study on the effects of hermetic storage on *Sitophilus oryzae* (L.) (Coleoptera: Curculionidae) provided information on the storage duration required for the cessation of *S. oryzae* activity [23]. Activity levels were determined using acoustic methods and mortality was established to occur at the 2% oxygen level, which required 3–14 d to achieve, depending on the initial infestation level and the initial volume of oxygen available to consume.

Acoustic detection has been used previously to test for the presence or absence of adult or larval infestation on stored grain by assessing insect activity through measurements of burst rates, burst impulse rates, and impulses per burst, where bursts are distinctive trains of sound impulses produced during insect activity, namely feeding, mating, locomotion, and egg-laying, that is rarely observed when insects are absent [24,25]. Similarly, a study on the detection of hidden infestations using adult *C. maculatus* on cowpea showed that feeding activity could be detected at 40 kHz using a “biomonitor” [26]. More recently, the acoustic biomonitor was also applied as a rapid method for testing cowpea seed resistance to *C. maculatus* among susceptible and resistant varieties [27]. 

This study employed acoustic methods in predicting when *C. maculatus* becomes inactive and ceases causing economic damage during hermetic storage. Oxygen depletion and acoustic activity were monitored for six different population levels and container size treatments of *C. maculatus* infestations on stored cowpea over a 15 d period.

## 2. Materials and Methods 

### 2.1. Insect Rearing and Grain Infestation

*Callosobruchus maculatus* adults in this experiment were originally collected in Niger, West Africa and were reared on cowpea (*Vigna unguiculata* (L.) Walp.) black-eyed southern peas (variety #8046) obtained from The Wax Company, LLC (Amory, MS, USA). They were reared at the Department of Entomology, Purdue University, in a Conviron^TM^ Environmental Chamber (C710, Winnipeg, MB, Canada) at 25 ± 1 °C; relative humidity 40 ± 5%; photoperiod 12:12 L:D. Cowpea grain for the experiment was disinfested for 14 d at −18 °C and then thawed at room temperature 1 d before the start of the experiment.

Colonies were set up approximately one month prior to the start of the experiment and the initial batch of emerging adults was sieved off with a number 10 sieve. After 48 h, emerged two-day old adults were isolated from those colonies using a number10 sieve. The experiments were carried out in three replications; therefore, six sets of 25, 50, and 100 *C. maculatus* adults were transferred via vacuum aspiration to nine 500 mL jars and nine 1000 mL jars containing clean uninfested cowpea. 

Data loggers (EL-USB-2, Lascar electronics Inc., Erie, PA, USA) were set in the experimental containers to record the temperature and RH every 30 min.

### 2.2. Acoustic Probe Installation

The experimental jars were spherical, reusable Pyrex^®^ 1000 mL and 500 mL glass jars (Corning Inc., Kaiserslautern, Germany) sealed with one-hole rubber stoppers (size number 7). The rubber stopper holes were drilled to accommodate a stainless steel probe using a cordless drill (Black & Decker (US), Towson, MD, USA) fitted with a 19/64-inch drill bit (Menards Inc., Eau Claire, WI, USA) [23].

The probe was fitted through the drilled hole in each rubber stopper. The probe served as a waveguide for the transmission of vibrational signals to the piezoelectric sensor/amplifier/recorder system. 

### 2.3. Oxygen Monitoring

Oxygen was monitored at the start of the experiment, twice daily (morning and evening) for the first six days, and twice a week for the next nine days. To enable this, two OxyDots (Oxysense Inc., Dallas, TX, USA) were attached on the inner walls of the hermetic jars. Oxygen levels were monitored using an Oxysense Portable Oxygen Analyzer (OxySense^®^, Dallas, TX, USA). The analyzer has a fiber optic reader-pen which illuminates the OxyDots and a photodetector which measures change in the intensity and fluorescence characteristics of the light reflected back from the OxyDot, which is related to the oxygen concentration inside the sealed jars.

### 2.4. Estimation of Available Oxygen in the Experimental Glass Jars 

To estimate the initial volumes of oxygen available for the insects to consume in the different-sized jars, we measured the volume of water needed to fill each grain-filled jar [23]. The volume of air available in the headspace and intergranular space of the cowpea-filled 1000 mL jars was found to be 435 mL, while that of the 500 mL jars was 237 mL. Because oxygen makes up about 21% of the atmospheric air, the oxygen available at the beginning of the experiment (*AO*) was estimated to be 50 mL and 92 mL in the 500- and 1000 mL jars, respectively.

### 2.5. Estimation of Available Oxygen and Initial Infestation Level in a PICS Bag 

The capacity of a 100 kg PICS bag was determined to be 135,000 mL [28]. The density of cowpea is 0.772 kg/liter [29] and therefore the volume of 100 kg cowpea was calculated to be 129.53 liters (129,530 mL). Therefore, the available air in a PICS bag filled with 100 kg cowpea was calculated as the difference in these two volumes, which is 5470 mL. Since air is 21% oxygen, the available oxygen in a 100 kg PICS bag was calculated to be 1149 mL.

Assuming an initial infestation for cowpea of eight insects/kg [30], it was determined that 100 kg of cowpea could have a total of 800 insects at the onset of storage. These values of estimated available oxygen and initial infestation were used to determine the time taken to reach the critical oxygen level where *C. maculatus* does not cause significant damage to stored cowpea, as explained in the discussion.

### 2.6. Acoustic Activity Monitoring

Immediately after filling and sealing the hermetic jars with cowpea and *C. maculatus* adults, and taking oxygen measurements, insect activity was monitored using acoustic methods. Early morning and late evening 1-h long recordings were taken for the first six days. For the next nine days, the recording frequency was reduced to once per day since the insect activity had decreased. The recordings were carried out in a secluded area with minimal background noise interference.

The setup for monitoring and recording insect signals was similar to that described previously for *Sitophilus oryzae* on stored wheat [23]. A piezoelectric sensor-preamplifier module (model SP-1L Acoustic Emission Consulting (AEC), Sacramento, CA, USA) was attached to the probe that passed through the stopper into the grain in the jars. The sensor module was connected to an AED 2010 amplifier (AEC, Sacramento, CA, USA). The AED-2010 was connected to a digital audio recorder, Marantz professional (model PMD-561, New York, NY, USA), which stored the insect signals as wav (wave audio–file-format) files on memory cards at a 44.1 kHz sampling rate. 

### 2.7. Signal Processing

Recorded signals were pre-screened using Raven Lite software [31], and a custom written insect signal analysis software program, DAVIS [32,33,34,35], performed analyses of a >60 s sample selected at random from each recording to distinguish insect sound impulses from occasionally occurring background noise. Movement and feeding sounds of insects in stored products are typically produced as trains of brief, 1–10-ms impulses separated by <200 ms intervals [24]. For each analyzed section, the DAVIS program classified these individual sound impulses as insect signals or background noise by least squares matching of the frequency spectrum of each impulse against previously determined spectral profiles of known insect sounds, i.e., summing the squared differences of the spectrum levels between the impulse and each profile [23,24]. If the sum of the squared differences was not less than a previously determined threshold value for at least one of the tested profiles, the impulse was classified as background noise and discarded from further analysis. In this experiment, we matched the impulse spectra against three different representative profiles, each obtained from recordings of separate infestations observed in a preliminary study. Impulse trains that contained at least three impulses which matched one of the three profiles were categorized as insect sound bursts [32,33,34,35,36]. The times and types of individual impulses and insect sound bursts were saved in a spreadsheet for analysis of mean burst rates, mean counts of impulses per burst, and mean rates of impulses in bursts [37].

### 2.8. Statistical Analysis

Analysis of variance (ANOVA) of oxygen concentration and consumption, mean rates of bursts, mean rates of burst impulses, and mean numbers of impulses per burst were conducted using Stata SE Version 12 [38]. Analysis of covariance (ANCOVA) was applied to test the effects of treatment, storage time, and their interaction. For insect activity, the coefficient of the interaction term was significant (*p* ≤ 0.001) and therefore one-way ANOVA was performed to test for daily differences during the first five days of storage. Means were separated using Bonferroni adjustment at the 95% confidence level. The regression of mean rates of bursts and mean rates of burst impulses on mean oxygen level was analyzed using Proc GLM, SAS Institute 2012 Version 9.4 [39]. 

## 3. Results

### 3.1. Oxygen Depletion Patterns and Rates

**Figure 1:**
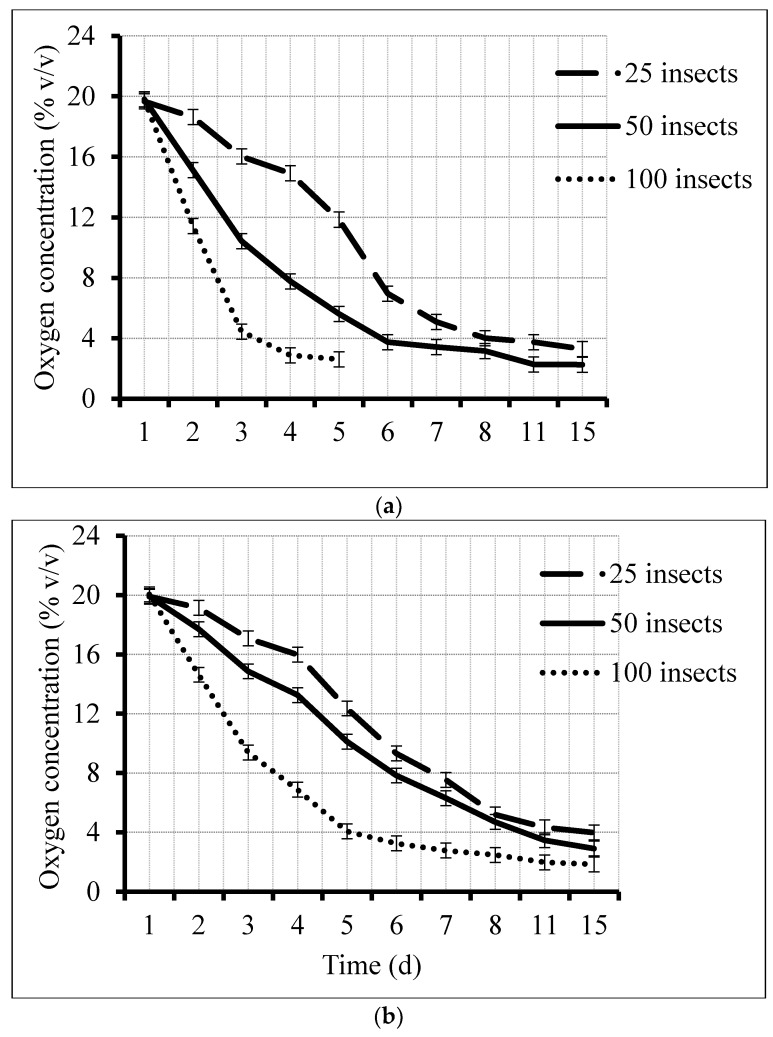
Oxygen level for 25, 50, and 100 *Callosobruchus maculatus* adults enclosed in hermetically sealed (**a**) 500 mL jars and (**b**) 1000 mL jars during a 15 d hermetic storage period.

Daily recorded oxygen levels show distinct decline patterns for the six treatments, depending on the original infestation level and oxygen volume (Figure 1). There were statistically significant effects of treatment (*F*_5503_ = 39.7, *p* < 0.001), storage time (*F*_8503_ = 104.4, *p* < 0.001), and their interaction (*F*_26,503_ = 11.28, *p* < 0.001). Further ANOVA analysis to test significant differences at different points in time showed that there were significant differences (*p* < 0.05) from day two through five (Table 1).

The time for the residual oxygen level in each treatment to decrease to 4% (*t*_4%_) was identified from the curves in Figure 1: 8, 6, and 3 d for the 25:500 mL, 50:500 mL, and 100:500 mL treatments, respectively; and 11, 7, and 5 d for the 25:1000 mL, 50:1000 mL, and 100:1000 mL treatments, respectively. The acoustic activity was minimal when the residual oxygen was less than 4%. 

**Figure 2:**
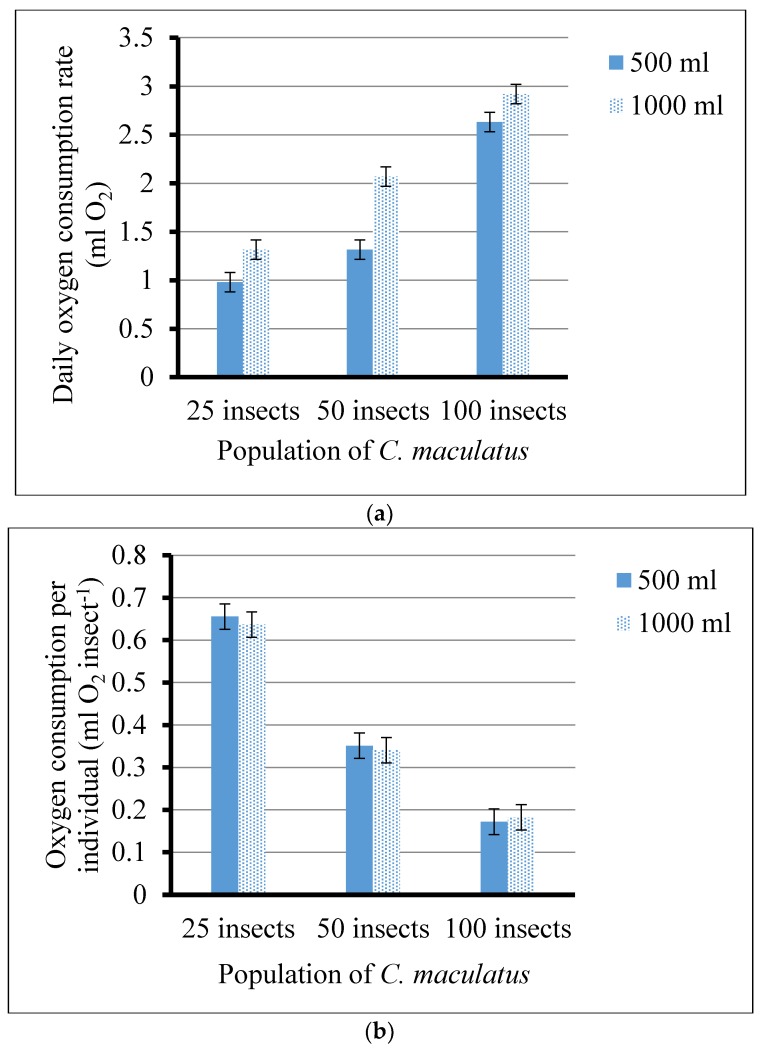
Measures of oxygen consumption during the time for the residual oxygen to deplete to 4%, *t*_4%_, after initiation of treatments: (**a**) Estimated daily O_2_ consumption for different treatments (Total mL O_2_ consumed/*t*_4%_); (**b**) Estimated individual O_2_ consumption (Total mL O_2_ consumed per insect)*.*

Daily oxygen consumption rates were calculated for each treatment (Figure 2A). It was of interest to also consider the mean individual consumption for the different population levels of *C. maculatus* (Figure 2B). The observed reduction in the oxygen consumption per individual, *IOC*, as the number of individuals per treatment increased (Figure 2B), suggested that the daily reductions in residual oxygen induced proportionate reductions in acoustic activity levels that would result in a statistically significant regression of activity on residual oxygen, presented later in Section 3.4 of this paper.

### 3.2. Temperature and Relative Humidity Variation

The experiments were carried out in a temperature controlled room at 25 °C and data loggers were inserted in the 25, 50, and 100 insects per 1000 mL jars treatments to record temperature and relative humidity for 15 days. The temperature exhibited cyclic variation ranging from 25.5 to 27.5 °C and there was no difference among the three treatments. The relative humidity ranged from 56–60%.

### 3.3. Acoustic Activity of C. maculatus during Hermetic Storage

**Figure 3:**
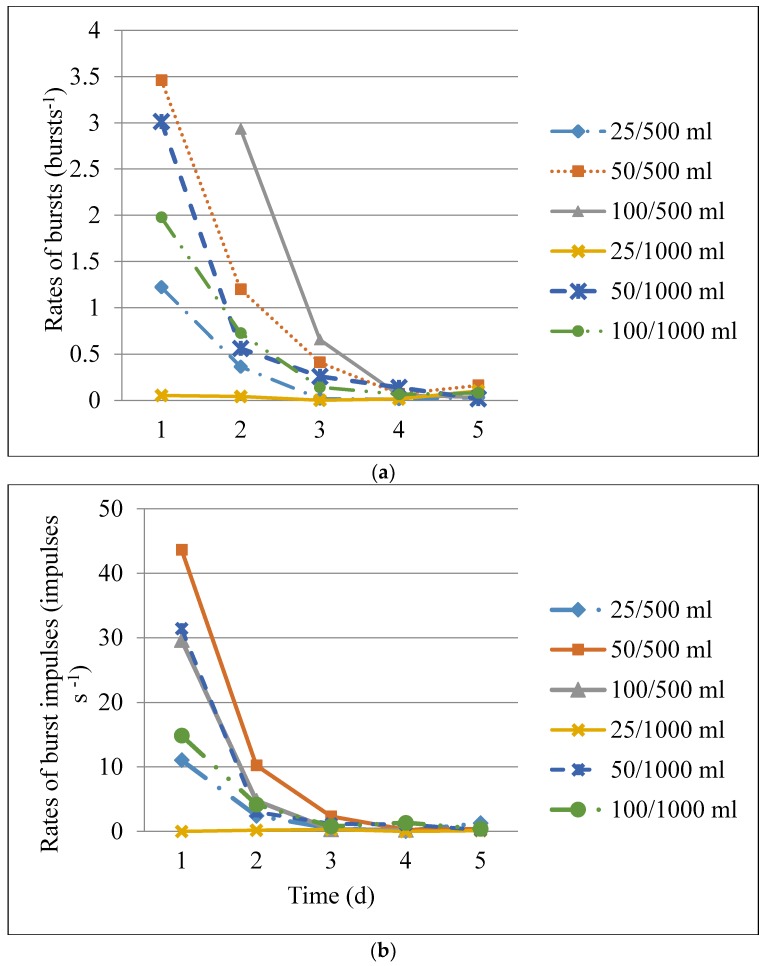
Burst rates (**a**) and rates of impulses in bursts (**b**) for 25, 50, and 100 *Callosobruchus maculatus* adults enclosed in hermetically sealed 500 mL jars and 1000 mL jars during the first 5 d of hermetic storage.

The rates of bursts and rates of impulses in bursts in the different *C. maculatus* treatments declined sharply over the first 5 d of the hermetic storage period (Figure 3). The decline was most drastic from day 1 to day 2, and then slowed down towards day 5 for all the treatments.

An ANCOVA analysis was performed to test whether there were significant differences in the decline curves of the different treatments and demonstrated a significant interaction between treatments and storage time, as shown in Table 2. 

An ANOVA analysis performed to test significant differences at different points in time showed that there was a significant difference (*p* < 0.05) among the treatments on the first four days when the insects were most active (Table 3).

### 3.4. Regression Analysis

Regression analysis was employed to consider *C. maculatus* acoustic activity levels per insect in relation to oxygen concentration depletion = (initial − residual) oxygen percentage from Figure 1. First, we examined the burst rate, rates of impulses in bursts, and impulses per burst in relation to oxygen depletion levels for the different treatments and determined that the curves for burst rate and rates of impulses in bursts were nonlinear. To linearize the relationship, the rates of bursts and rates of impulses in bursts were divided by the number of insects in each treatment, *N_t_*, and then transformed as Log_10_ (magnitude +1). This resulted in regression equations for each treatment that generally provided the line of best fit for population levels examined when the *B_r_* = Log_10_ (burst rate/*N_t_*+ 1), *I_r_* = Log_10_ (rate of impulses in bursts/*N_t_* + 1), and *I_pb_* = numbers of impulses per burst were plotted against the oxygen concentration depletion level (*R_o_*) according to Equations (1)–(3) below.

*B_r_* = *intercept*_1_ + *m*_1_*Ro* (1)

*I_r_* = *intercept*_2_ + *m*_2_*Ro* (2)

*I_pb_* = *intercept*_3_ + *m*_3_*Ro* (3)

The intercepts and slopes obtained from regression analysis of the activity of the different *C. maculatus* population levels on oxygen concentration depletion level for all the treatments are shown in Table 4.

**Figure 4:**
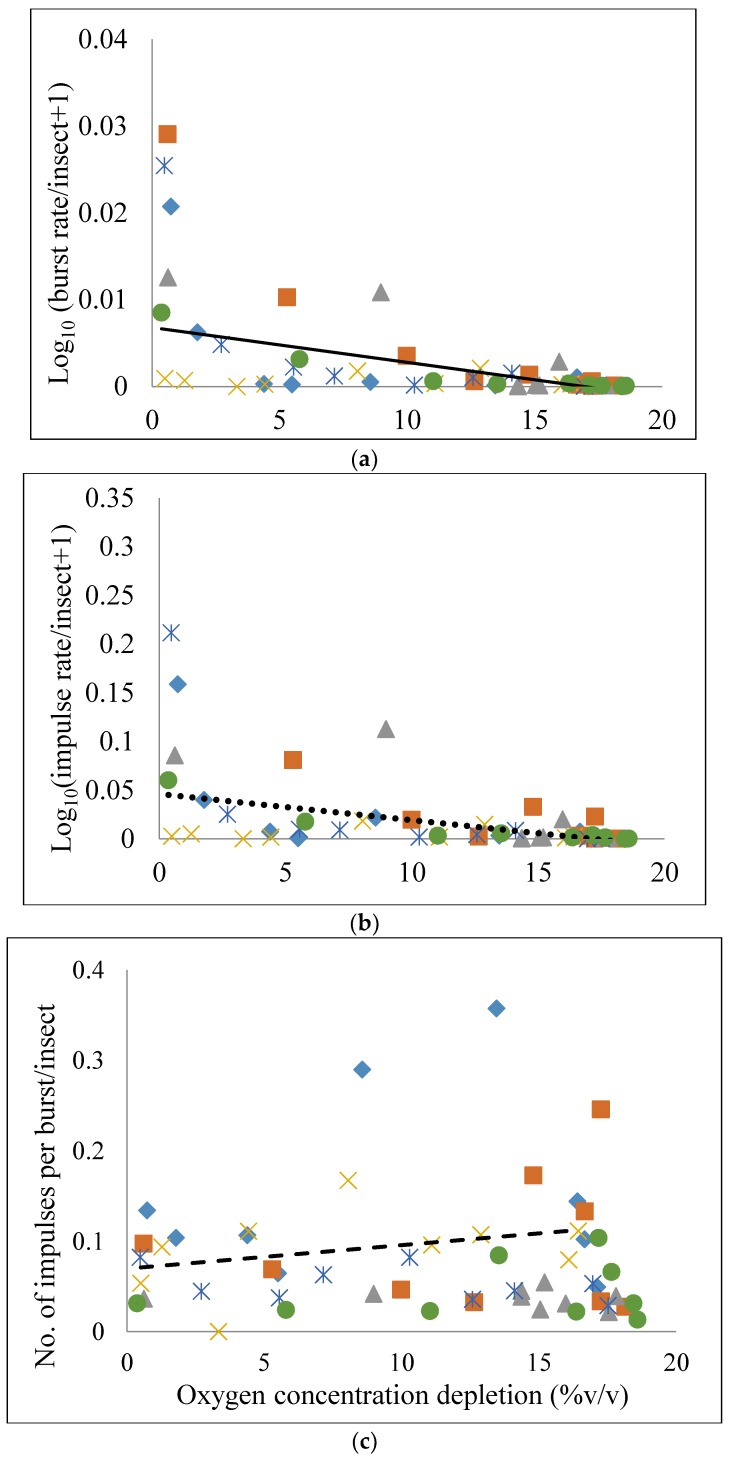
Scatter plot showing the relationship of (**a**) log_10_(burst rate/insect + 1); (**b**) log_10_ (rate of impulses in bursts/insect + 1); and (**c**) Number of impulses per burst/insect to the oxygen concentration depletion for 25 *Callosobruchus maculatus* adults in 500 mL (diamond) and 1000 mL (X) storage jars, 50 adults in 500 mL (square) and 1000 mL (asterisk) storage jars, and 100 adults in 500 mL (triangle) and 1000 mL (circle) storage jars during a 15 d storage period.

The models for Equations (1) and (2) are statistically significant, with *F*_1.52_ = 27.71 (*p* < 0.001) with *R*^2^ = 0.348 for Equation (1), and *F*_1.52_ = 21.33 (*p* < 0.001) with *R*^2^ = 0.291 for Equation (2). However, the model for Equation (3) was not statistically significant with *F*_1.52_ = 1.70 (*p* = 0.1976) with *R*^2^ = 0.0317. The intercepts and slopes of the regression equations are significantly different from zero [*p* (>*t*) < 0.05] (Table 4). The regression lines are shown in Figure 4A (solid line), Figure 4B (dotted line), and Figure 4C (dashed line). As expected, the values of *B_r_*, and *I_r_* all decreased with decreasing residual O_2_ percentage.

## 4. Discussion

In this study, we assessed how the activity of three different population levels of *C. maculatus* infesting hermetically sealed cowpea decreased over time as the oxygen level decreased. As noted in Jalinas [37], reduced levels of feeding and movement of insects under oxygen stress [40] are expected to reduce both the sound burst rate and the rate of impulses in bursts. Our results demonstrated that insects in all treatments ceased acoustic activity and became quiescent, i.e., ceased feeding, ovipositing, and other movements when O_2_ decreased below 4% during hermetic storage. From an economic perspective, insect quiescence in this study is similar to insect mortality in other studies of oxygen deprivation because the insects no longer cause damage to the stored product, even if they briefly continue to remain alive. Quiescence in response to oxygen deprivation was achieved within one week of sealing infested cowpea grain in the hermetic containers, and in a similar study examining mortality in response to oxygen deprivation in hermetic containers, the oxygen level declined from 19.2% to 2.3% within five to seven days, resulting in the death of 50 and 200 *C. maculatus* adults in 210 and 850 mL jars, respectively [7]. The specific lethal effect was reported as 91.1% adult mortality after four days and 100% from the fifth day [7].

The results of this study show that the initial infestation level and initial quantity of oxygen available dictated the susceptibility and survival duration of *C. maculatus* as O_2_ levels decreased. Similar studies on hermetic storage have shown that the depletion rate of oxygen depends on the duration of storage, size of storage container, the developmental stage of the insects, and the level of infestation present [23,41]. Individual consumption rates for the *C. maculatus* in this study also depended on the initial population level and the available oxygen. This was clearly demonstrated by the individual oxygen consumption rates for the same population level in 1000 mL treatments, which contained double the initial oxygen volume of 500 mL treatments. This scenario was also observed in a previous study with *S. oryzae* [23], whereby higher population levels drove oxygen down faster in the smaller jars (100 insects per 500 mL) as compared to smaller population levels in the larger jars (25 insects per 1000 mL). However, individual insect oxygen consumption showed no significant difference between the two volumes (1000 mL vs. 500 mL) for the same population level. 

Other studies have assessed the individual insect consumption rates at normoxic conditions and found that approximately 8 mL O_2_ is needed during the lifetime of *C. maculatus* adults (egg to adult emergence) [14,42,43]. Our findings focused on adult stages under hypoxic conditions which prompted the insects to reduce their metabolic activities. The individual insect oxygen consumption (IOC) per day was between 0.17 for 100 insects per 500 mL to 0.66mL for 25 insects per 500 mL (Figure 2B) over a 3–11 d period before the achievement of *t*_4%_. While the insects lived for about five days under these low oxygen conditions [14], the available oxygen per adult was not sufficient to complete the entire life cycle of the subsequent generations and therefore after incubation of all treatments for 45 days, there were no emerging insects. This is important in hermetic storage where farmers store pre-infested grain in sealed bags. The available oxygen is usually not sufficient to produce future generations and as such, the stored grain is saved from further deterioration.

The low oxygen levels under hermetic conditions result in the suppression of feeding, growth, development, and population expansion [13,14,43,44]. In addition, before the insects succumb, they respond to hypoxia by metabolic down-regulation which reduces insect movement overall. The behavioral effects of these changes can be monitored using acoustic means to show when insect quiescence occurs. Therefore, this study found that the different population levels of *C. maculatus* produced sound impulses with a broad range of amplitudes, spectral features, and temporal patterns, and which were comparable with those observed for other stored product pests such as *Prostephanus truncatus* (Coleoptera: Bostrichidae), *Sitophilus zeamais* (Coleoptera: Curculionidae), *Acanthoscelides obtectus* (Coleoptera: Chrysomelidae: Bruchinae), and *Sitophilus oryzae* (Coleoptera: Curculionidae) [29,30,31,37].

The acoustic patterns of *C. maculatus* observed in this study are similar to those observed in *S. oryzae* during hermetic storage [23]. An important observation was the cessation of activity at the 4% oxygen level after 3–11 d exposure to hermetic storage conditions. Also of interest was the decline of burst rates after one week of storage to below 0.02 bursts s^−1^, which has previously been determined to be the threshold below which a low likelihood of infestation is predicted acoustically [24] and, in this study, is associated with the 4% oxygen level at which quiescence occurred. Studies have shown that insects cope with low oxygen and elevated carbon dioxide by reducing their metabolism, thus reducing ATP (Adenosine triphosphate) and energy accessibility [45]. This reduced energy resulted in concomitant declines in the rates of individual movements (burst rate) and the duration of movement (rate of impulses in bursts)*.*

The increasing use of hermetic technologies such as PICS bags as an inexpensive, effective way to control storage insect pests in Africa and Asia [46,47,48] warrants studies to understand insect behavior in these sealed environments. Additionally, given that multiple studies have found that hermetic storage causes insect mortality at oxygen levels below 5% [7,23], two important concerns for the practical implementation of hermetic storage technologies are: (1) the typical oxygen level reductions achieved in hermetic storage bags in field environments; and (2) the exposure duration required at such levels to cause total mortality or reach a quiescence threshold where the insects will not cause any further damage to the grain. This exposure duration, *t*_4%_, can be estimated for different values of initial available oxygen (*AO*) volume and different numbers of insects per treatment, *N_t_*, by considering the ranges of individual oxygen consumption (*IOC*) found in Figure 2B and applying a simple equation that approximates their interrelationships:

*t*_4%_ = *AO*/(*N_t_ IOC*) (4)

As an example of how effectively the approximation reproduces the measured value of *t*_4%_, consider the treatment where oxygen was reduced at the lowest rate, with 25 insects in a 1000 mL hermetic container, in which *t*_4%_ was measured as 11 d (Figure 1B). Calculated *AO* in 1000 mL and 500 mL jars of cowpea are 92 mL and 50 mL, respectively. The values of *IOC* in Figure 2B ranged from 0.167 to 0.66, with a midpoint of 0.41 mL O_2_/d. Applying these three estimates of individual oxygen consumption per day in the calculations, 92/(25 · *IOC*), yields *t*_4%_ estimates of 22, 5.6, and 9.0 d, respectively. Similarly, consider the treatment where oxygen was reduced at the highest rate, with 100 insects in a 500 mL hermetic container in which *t*_4%_ was measured as 2.6 d (Figure 1A). Applying the same *IOC* estimates in the calculations, 50/(100 · *IOC*), yields *t*_4%_ estimates of 2.99, 0.75, and 1.21 d, respectively. In both cases, the actual measurement occurred within the overall range of *IOC* measurements. Now consider that the initial *AO* volume of a 100 kg PICS bag is 1149 mL O_2_, and a typical count of insects in one of these bags could be 800 (8 adults per kg). Typical values of *t*_4%_ expected for a PICS bag would then be 1149/(800 · *IOC*), or 8.45, 3.50, and 2.18 d, respectively.

## 5. Conclusions

Overall, the relationship between individual insect oxygen consumption of the insects (*IOC*), available oxygen (*AO*), and starting population level (*N_t_*) before hermetic storage could be beneficial in understanding how fast oxygen is depleted to levels where insects can no longer damage the grain. Acoustic activity cessation and overall quiescence of insects during hermetic storage occurs at oxygen levels below 4% and is a good indicator of the safety level of stored grain. Knowledge of the dynamics within the hermetic enclosures heightens the appeal to use hermetic storage as opposed to conventional non-hermetic storage methods.

## Figures and Tables

**Table 1:** Mean (±SE) residual oxygen level for 25, 50, and 100 adults of *Callosobruchus maculatus* on days one to five of hermetic storage treatment in 500 mL and 1000 mL jars (*n* = 396) .

Treatment	Residual Oxygen Level (%) *				
	Day 1	Day 2	Day 3	Day 4	Day 5
25 insects/1000 mL	19.91 ± 0.04 a	19.14 ± 0.31 a	17.08 ± 0.25 a	15.98 ± 0.28 a	12.35 ± 0.79 a
25 insects/500 mL	19.68 ± 0.77 a	18.63 ± 0.97 ab	16.02 ± 1.10 ab	14.92 ± 2.24 ab	11.85± 3.37 ab
50 insects/1000 mL	19.93 ± 0.08 a	17.71 ± 0.72 b	14.86 ± 0.39 b	13.26 ± 0.36 b	10.12± 0.52 b
50 insects/500 mL	19.80 ± 0.34 a	15.12 ± 1.56 c	10.40 ± 1.16 c	7.76 ± 0.94 c	5.61 ± 1.02 c
100 insects/1000 mL	20.05 ± 0.14 a	14.63 ± 1.38 c	9.37 ± 0.53 c	6.87 ± 0.87 c	4.06 ± 0.63 c
100 insects/500 mL	19.79 ± 0.66 a	11.43 ± 1.74 d	4.44 ± 1.56 d	2.86 ± 1.03 d	2.61 ± 1.30 d

* All data are means ± SEM. Entries in the same column followed by same letters are not significantly different (*p* ≥ 0.05). Means were separated using Bonferroni adjustment.

**Table 2:** Analysis of the effects of hermetic storage treatment, storage time, and their interaction on the mean rates of bursts, rates of impulses in bursts, and number of impulses per burst (*n* = 630 observations).

Parameter	Df	*F*	*p*
Rates of bursts			
Treatment	4	15.48	<0.001
Storage time	8	23.29	<0.001
Treatment × Storage time	22	5.34	<0.001
Rates of impulses in bursts			
Treatment	4	13.36	0.0008
Storage time	8	16.97	0.0203
Treatment × Storage time	22	4.55	0.0033
Impulses per burst			
Treatment	4	3.96	<0.001
Storage time	8	1.73	<0.001
Treatment × Storage time	22	2.06	<0.001

**Table 3:** Analysis of variance of insect sound burst rates produced by 25, 50, and 100 *Callosobruchus maculatus* adults during the first five days of hermetic storage treatment in 500 mL and 1000 mL jars (*n* = 324).

Treatment	Daily *Callosobruchus maculatus* Mean Activity (Bursts s^−1^) *				
	Day 1	Day 2	Day 3	Day 4	Day 5
25 insects/500 mL	0.40 ± 0.12 a	0.12 ± 0.03 a	0.02 ± 0.01 a	0.0 ± 0.0 a	0.01 ± 0.01 a
25 insects/1000 mL	0.02 ± 0.01 b	0.01 ± 0.004 b	0.0 ± 0.0 a	0.01± 0.001 a	0.03 ± 0.02 a
50 insects/500 mL	1.15 ± 0.22 ac	0.40 ± 0.07 c	0.14 ± 0.03 b	0.02 ± 0.004 b	0.05 ± 0.01 a
50 insects/1000 mL	1.01 ± 0.20 c	0.19 ± 0.06 a	0.09 ± 0.02 ab	0.05 ± 0.02 b	0.01 ± 0.003 a
100 insects/500 mL	0.83 ± 0.19 ac	0.98 ± 0.14 a	0.22 ± 0.03 c	0.02 ± 0.01 b	0.01 ± 0.003 a
100 insects/1000 mL	0.65 ± 0.19 ac	0.24 ± 0.04 a	0.05 ± 0.02 ab	0.02 ± 0.01 b	0.03 ± 0.01 a

* All data are means ± SEM. Entries in the same column followed by same letters are not significantly different (*p* ≥ 0.05). Means were separated using Bonferroni adjustment.

**Table 4:** Intercepts and slopes (±SEM) for regression equations (all values × 10^−3^) for regression equations fitting the models in Equations (1)–(3) for 25, 50, and 100 adult *C. maculatus* hermetically enclosed in 500 mL and 1000 mL hermetic storage treatments.

Measurement	Intercept ± SEM	*t*	*p > t*	Slope ± SEM	*t*	*p > t*
*B_r_* (Equation (1))	9.44 ± 1.412	6.69	<0.001	−0.585 ± 0.111	−5.27	<0.001
*I_r_* (Equation (2))	75.33 ± 12.62	5.97	<0.001	−4.598 ± 0.993	−4.62	<0.001
*I_pb_* (Equation (3))	7075.6 ± 2097	3.37	0.0014	215.5 ± 165.13	1.30	0.1976

## References

[1] Nedumaran S., Abinaya P., Jyosthnaa P., Shraavya B., Rao P., Bantilan C. (2015). Grain Legumes Production, Consumption and Trade Trends in Developing Countries.

[2] Singh R.J. (2006). Genetic Resources, Chromosome Engineering, and Crop Improvement: Vegetable Crops.

[3] Langyintuo A.S., Lowenberg-DeBoer J., Faye M., Lambert D., Ibro G., Moussa B., Ntoukam G. (2003). Cowpea supply and demand in West and Central Africa. Field Crops Res..

[4] Carlos G. (2000). Cowpea: Post Harvest Operation.

[5] Hill S.B. (1989). Pest Control—Cultural Control of Insects. Cultural Methods of Pest, Primarily Insect Control.

[6] Mbata G.N., Hetz S.K., Reichmuth C., Adler C. (2000). Tolerance of pupae and pharate adults of *Callosobruchus subinnotatus* Pic (Coleoptera: Bruchidae) to modified atmospheres: A function of metabolic rate. J. Insect Physiol..

[7] Seck D., Lognay G., Haubruge E., Marlier M., Gaspar C. (1996). Alternative protection of cowpea seeds against *Callosobruchus maculatus* (F.) (Coleoptera: Bruchidae) using hermetic storage alone or in combination with *Boscia senegalensis* (Pers.) Lam ex Poir. J. Stored Prod. Res..

[8] Ntoukam G., Murdock L.L., Shade R.E., Kitch L.W., Endondo C., Ousmane B., Wolfson J. Managing insect Pests of cowpea in storage. Proceedings of the Midcourse 2000 Research Meeting of Bean/Cowpea.

[9] Johnson J.A., Valero K.A. (2003). Use of Commercial Freezers to Control Cowpea Weevil, *Callosobruchus maculatus* (Coleoptera: Bruchidae), in Organic Garbanzo Beans. J. Econ. Entomol..

[10] Baoua I.B., Margam V., Amadou L., Murdock L.L. (2012). Performance of Triple Bagging Hermetic Technology for Postharvest Storage of Cowpea Grain in Niger. J. Stored Prod. Res..

[11] Bailey S.W. (1955). Air-Tight Storage of Grain; Its Effects on Insect Pests. I. *Calandra granaria* L. (Coleoptera, Curculionidae). Aust. J. Agric. Res..

[12] Murdock L.L., Baoua I.B. (2014). On Purdue Improved Cowpea Storage (PICS) technology: Background, mode of action, future prospects. J. Stored Prod. Res..

[13] Baoua I.B., Amadou L., Baributsa D., Murdock L.L. (2014). Triple bag hermetic technology for post-harvest preservation of Bambara groundnut (*Vigna subterranea* (L.) Verdc.). J. Stored Prod. Res..

[14] Murdock L.L., Margam V., Baoua I., Balfe S., Shade R.E. (2012). Death by desiccation: Effects of hermetic storage on cowpea bruchids. J. Stored Prod. Res..

[15] Baributsa D., Fletcher-Timmons H. (2017). Purdue Improved Crop Storage (PICS) Newsletter.

[16] Hashem M.Y., Risha E.S.M., El-Sherif S.I., Ahmed S.S. (2012). The effect of modified atmospheres, an alternative to methyl bromide, on the susceptibility of immature stages of angoumois grain moth *Sitotroga cerealella* (Olivier) (Lepidoptera: Gelechiidae). J. Stored Prod. Res..

[17] Mbata G.N., Phillips T.W. (2001). Effects of temperature and exposure time on mortality of stored-product insects exposed to low pressure. J. Econ. Entomol..

[18] Navarro S., Dias R., Donahaye E. (1985). Induced tolerance of *Sitophilus oryzae* adults to carbon dioxide. J. Stored Prod. Res..

[19] Ofuya T.I., Reichmuth C. (2002). Effect of relative humidity on the susceptibility of *Callosobruchus maculatus* (Fabricius) (Coleoptera: Bruchidae) to two modified atmospheres. J. Stored Prod. Res..

[20] Yan Y., Williams S.B., Murdock L.L., Baributsa D. (2017). Hermetic storage of wheat and maize flour protects against red flour beetle (*Tribolium castaneum* Herbst). PLoS ONE.

[21] Cheng W., Lei J., Fox C.W., Johnston J.S., Zhu-Salzman K. (2015). Comparison of life history and genetic properties of cowpea bruchid strains and their response to hypoxia. J. Insect Physiol..

[22] Adler C., Corinth H.G., Reichmuth C. (2000). Modified atmospheres. Alternatives to Pesticides in Stored-Product IPM.

[23] Njoroge A.W., Mankin R.W., Smith B.W., Baributsa D. (2017). Effects of Hermetic Storage on Adult *Sitophilus oryzae* L. (Coleoptera: Curculionidae) Acoustic Activity Patterns and Mortality. J. Econ. Entomol..

[24] Mankin R.W., Mizrach A., Hetzroni A., Levsky S., Nakache Y., Soroker V. (2008). Temporal and spectral features of sounds of wood-boring beetle larvae: Identifiable patterns of activity enable improved discrimination from background noise. Fla. Entomol..

[25] Kiobia D.O., Tumbo S.D., Cantillo J., Rohde B.B., Mallikarjunan P.K., Mankin R.W. (2015). Characterization of sounds in maize produced by internally feeding insects: Investigations to develop inexpensive devices for detection of *Prostephanus truncatus* (Coleoptera: Bostrichidae) and *Sitophilus zeamais* (Coleoptera: Curculionidae) in small-scale storage facilities in sub-Saharan Africa. Fla. Entomol..

[26] Shade R.E., Furgason E.S., Murdock L.L. (1990). Detection of hidden insect infestations by feeding-generated ultrasonic signals. Am. Entomol..

[27] Devereau A.D., Gudrups I., Appleby J.H., Credland P.F. (2003). Automatic, rapid screening of seed resistance in cowpea, *Vigna unguiculata* (L.) Walpers, to the seed beetle *Callosobruchus maculatus* (F.) (Coleoptera: Bruchidae) using acoustic monitoring. J. Stored Prod. Res..

[28] Baributsa D., Baoua I., Murdock L.L. (2013). Purdue Improved Crop Storage (PICS) Bag: Size Matters.

[29] Carter S., Gartner S., Haines M., Olmstead A., Sutch R., Wright G. (2006). Historical Statistics of the United States, Millennial Edition.

[30] Silim Nahdy M., Ellis R.H., Silim S.N., Smith J. (1998). Field infestation of pigeonpea (*Cajanus cajan* (L.) Millsp.) by *Callosobruchus chinensis* (L.) in Uganda. J. Stored Prod. Res..

[31] Bioacoustics Research Program (2016). Raven Lite: Interactive Sound Analysis Software.

[32] Njoroge A.W., Affognon H., Mutungi C., Richter U., Hensel O., Rohde B., Mankin R.W. (2017). Bioacoustics of *Acanthoscelides obtectus* (Coleoptera: *Chrysomelidae*: Bruchinae) on *Phaseolus vulgaris* (Fabaceae). Fla. Entomol..

[33] Njoroge A.W., Affognon H., Mutungi C., Rohde B., Richter U., Hensel O., Mankin R.W. (2016). Frequency and time pattern differences in acoustic signals produced by *Prostephanus truncatus* (Horn) (Coleoptera: Bostrichidae) and *Sitophilus zeamais* (Motschulsky) (Coleoptera: Curculionidae) in stored maize. J. Stored Prod. Res..

[34] Mankin R.W., Brandhorst-Hubbard J., Flanders K.L., Zhang M., Crocker R.L., Lapointe S.L., Weaver D.K. (2000). Eavesdropping on insects hidden in soil and interior structures of plants. J. Econ. Entomol..

[35] Herrick N.J., Mankin R.W., Dosunmu O.G., Kairo M.T.K. Ecology and detection of the red palm weevil, *Rhynchophorus ferrugineus* (Coleoptera: Curculionidae), and related weevils for the protection of palm tree species in the United States. Proceedings of the Colloque Méditerranéen sur les Ravageurs des Palmiers.

[36] Mankin R.W., Hagstrum D.W., Smith M.T., Roda A.L., Kairo M.T.K. (2011). Perspective and promise: A century of insect acoustic detection and monitoring. Am. Entomol..

[37] Jalinas J., Güerri-Agulló B., Mankin R.W., López-Follana R., Lopez-Llorca L.V. (2015). Acoustic assessment of *Beauveria bassiana* (Hypocreales: Clavicipitaceae) effects on *Rhynchophorus ferrugineus* (Coleoptera: Dryophthoridae) larval activity and mortality. J. Econ. Entomol..

[38] StataCorp (2011). Stata Statistical Software: Release 12.

[39] SAS Institute Inc. (2013). Base SAS^®^ 9.4. Procedures Guide: Statistical Procedures.

[40] Jalinas J., Güerri-Agulló B., Dosunmu O.G., Lopez Llorca L.V., Mankin R.W. (2017). Acoustic activity cycles of *Rhynchophorus ferrugineus* (Coleoptera: Dryophthoridae) early instars after *Beauveria bassiana* (Hypocreales: Clavicipitaceae) treatments. Ann. Entomol. Soc. Am..

[41] Ofuya T.I., Reichmuth C. (1994). Effect of level of seed infestation on mortality of larvae and pupae of *Callosobruchus maculatus* (F.) (Coleoptera: Bruchidae) in some controlled atmospheres. J. Stored Prod. Res..

[42] Quellhorst H.E., Williams S.B., Murdock L.L., Baributsa D. (2018). Cumulative oxygen consumption during development of two postharvest insect pests: *Callosobruchus maculatus* Fabricius and *Plodia interpunctella* Hübner. J. Stored Prod. Res..

[43] Williams S.B., Murdock L.L., Kharel K., Baributsa D. (2016). Grain size and grain depth restrict oxygen movement in leaky hermetic containers and contribute to protective effect. J. Stored Prod. Res..

[44] Yan Y., Williams S.B., Baributsa D., Murdock L.L. (2016). Hypoxia Treatment of *Callosobruchus maculatus* Females and Its Effects on Reproductive Output and Development of Progeny Following Exposure. Insects.

[45] Mitcham E., Martin T., Zhou S. (2006). The mode of action of insecticidal controlled atmospheres. Bull. Entomol. Res..

[46] Jones M., Alexander C., Lowenberg-DeBoer J. (2011). Profitability of hermetic Purdue Improved Crop Storage (PICS) Bags for African Common Bean Producers.

[47] Baributsa D., Lowenberg-DeBoer J., Murdock L., Moussa B. Profitable chemical-free cowpea storage technology for smallholder farmers in Africa: Opportunities and challenges. Proceedings of the 10th International Working Conference on Stored Product Protection.

[48] Baributsa D., Abdoulaye T., Lowenberg-DeBoer J., Dabiré C., Moussa B., Coulibaly O., Baoua I. (2014). Market building for post-harvest technology through large-scale extension efforts. J. Stored Prod. Res..

